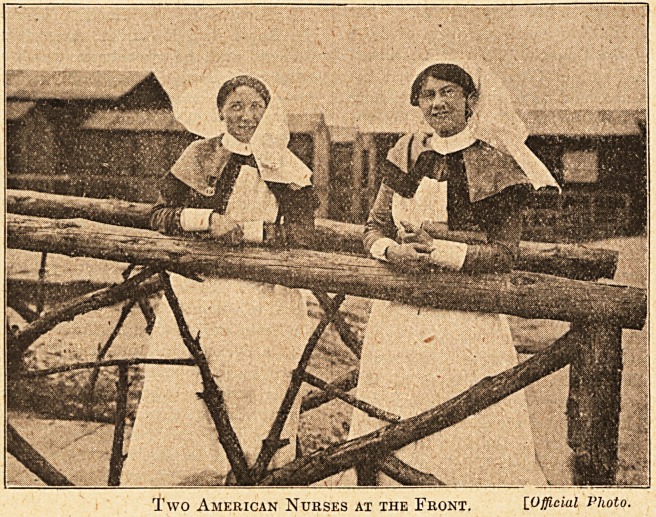# Round the Hospitals

**Published:** 1917-07-07

**Authors:** 


### ROUND THE HOSPITALS.

WE have received the second Annual Report of the College of Nursing, Limited, attached to which are the reports of the Scottish and Irish Boards and of the Treasurer: The Income and Expenditure Account of the College of Nursing, Limited, from March 27, 1916, to March 31, 1917, the College Balance-sheet to March 31, 1917, and the Scottish Board's Income and Expenditure Account from November 1, 1916, to March 31, 1917, and its Balance-sheet to March 31, all duly audited, are also attached. On pages 283 *et seq.* will be found the reports in full (*a*) of the College of Nursing, Limited, (*b*) of the Scottish Board, and (*c*) of the Treasurer. The Irish Board was only appointed in February last. Its report states it has held two meetings, that offices have been opened, a Secretary appointed, and fifty-two applications for registration have been received from nurses trained in Ireland. Although the Irish Board has "much bitter opposition to overcome and certain curious conditions peculiar to this country to be dealt with," it regards as an encouraging sign the facts that one of the best Dublin hospitals has arranged for its nurses from henceforth to have three years' instead of two years' consecutive work in the wards, and that a prominent Women's and Children's Hospital in the North has expressed a strong desire for affiliation with a good general hospital. The Report is signed by the Chairman of the Irish Board, Mr. George Peacocke, M.D., F.R.C.P.I., Dublin. The Annual Meeting of the College of Nursing, Limited, will be held at the Royal Society of Medicine, 1 Wimpole Street, London, W., on Thursday, 12th instant, at 3 P.M. We are glad to note that the report of the College of Nursing emphasises the importance of the valuable help the Council has received from British trained nurses who are engaged at the Front, and also from those working in the homeland and British Dominions. We may add that the Council has written under the date of 25th ult. to thank *The Nursing Mirror* and its Editor for the publicity that paper has given to the aims and objects of the College and for the substantial help its readers have rendered as recruiting sergeants, which has resulted in the addition to the membership of the College of two thousand nurses.

---

WITH reference to the paragraph in the *Poor-Law Officers' Journal* with regard to nominees for the Council of the College of Nursing, we are informed that no Association has any right to nominate members to the Council of the College; they may suggest names, but the Council alone has the right to appoint.

---

AN important development has taken place with regard to schools of massage in London. Hitherto there has been a school of massage in connection with the University College Hospital and one with the National Hospital for the Paralysed, Queen Square. The authorities of these hospitals have now appointed a joint committee to manage the school of massage situated in Queen Square, Bloomsbury, under the name of the National Hospital and University College Hospital School of Massage and Electrical Treatment. The first term is planned to commence early in October, and the curriculum will include the teaching of massage, remedial exercises, and medical electricity. This school will have the advantage of a hostel in connection with it, where students who desire special accommodation can reside in comfort.

For British Nurses and Nursing, see p. 278.

We have the pleasure of publishing this week a photograph showing two American nurses at the Front, with their uniform. They are members of the first American Unit that were received by their Majesties the King and Queen at Buckingham Palace, who greeted them as the first detachment of the American Army which has landed on our shores since their great Republic resolved to struggle for the ideals of civilisation. The whole nation endorses His Majesty's declaration that "it was characteristic of the "humanity and chivalry which has ever been "imaged by the American nation that the first "assistance rendered to the Allies is in connection "with the profession of healing and the "profession of "nursing." Queen Alexandra, the devoted and untiring friend of all nurses, received our American cousins at Marlborough House. The matrons in charge of the Harvard Unit, Miss Carrie M. Hall and Miss Hanna S. Peterson, with two other nurses, were received by H.R.H. the Duke of Connaught, who, as England's representative in the Dominion of Canada, has learnt their value and efficiency.

Two American Nurses at the Front. [Official Photo.

## Figures and Tables

**Figure f1:**